# Association Between Physician Communication Features and Patient Outcomes in Telemedicine: Retrospective Cross-Sectional Observational Study

**DOI:** 10.2196/86977

**Published:** 2026-03-26

**Authors:** Yipei Wang, Shu Wang, Ke Zhang, Zhijie Liu, Qingbian Ma, Hong Ji, Zheng Hou, Tracy Xiao Liu, Xuedong Xu, Xinxia Wu, Changxiao Jin

**Affiliations:** 1 Department of Medical Affairs, Office of Internet Hospital, Peking University Third Hospital, Beijing, China; 2 Institute of Hospital Management, Peking University Third Hospital, 49 North Huayuan Road, Haidian District, Beijing, 100191, China, 86 01082266191; 3 Department of Economics, School of Economics and Management, Tsinghua University, Beijing, China; 4 Department of Otolaryngology, Peking University Third Hospital, Beijing, China; 5 School of Computer Science and Technology, Beijing Institute of Technology, Beijing, China; 6 Department of Emergency Medicine, Peking University Third Hospital, Beijing, China; 7 Information Management and Big Data Center, Peking University Third Hospital, Beijing, China; 8 Department of Obstetrics and Gynecology, Peking University Third Hospital, Beijing, China; 9 Peking University People's Hospital, Beijing, China

**Keywords:** telemedicine, asynchronous visits, physician-patient communication, patient loyalty, patient satisfaction, behavioral pattern, quality of virtual care, health-seeking behavior

## Abstract

**Background:**

Asynchronous telemedicine is a crucial component of multichannel health care, where effective communication drives satisfaction. However, the effectiveness of communication features remains poorly understood. Prior research relied on subjective surveys or small-scale simulations, failing to link features to objective outcomes. Understanding these features is critical for optimizing physician engagement and establishing quality indicators to enhance the patient experience.

**Objective:**

This study aimed to bridge this gap by leveraging a large-scale real-world dataset to quantify the association between physicians’ communication features—including response modalities, length, and sequence—and patient repurchase behavior, as well as review scores, within a high-autonomy health care setting.

**Methods:**

This retrospective cross-sectional study analyzed 304,337 paid, patient-initiated virtual visits from a Chinese academic medical center (2021‐2023), which included 823,135 physician responses. The sample was selected after applying a series of exclusion criteria, such as free consultations, team-based visits, and outlier data. The key exposures were the modality of physician responses, response length, and response sequence. Outcomes included patient loyalty and satisfaction. Loyalty was operationalized as follow-up visits within 6 months, with a 30-day exclusion period applied to same-physician (*fv1*) and same-department (*fv2*) revisits to filter out clinical necessity, but not to hospital-wide revisits (*fv3*). Satisfaction was measured by the review scores. We used probit and ordinary least squares regressions to examine the relationships between communication features and patient outcomes.

**Results:**

Regarding loyalty, audio-only visits were associated with the lowest *fv1*, with an average marginal effect (AME) of −0.030 (95% CI −0.043 to −0.016, *P*<.001), translating to a 30.9% (0.030/0.097) reduction compared to text-only visits. Regarding satisfaction, audio messages were associated with a significantly increased likelihood of patients providing reviews, with an AME of 0.041 (95% CI 0.006‐0.076, *P*=.02), but they did not affect review scores after adjusting for inverse Mills ratios. Increased numbers of text and audio replies were (marginally) associated with improved *fv1*, with AMEs of 0.009 (95% CI 0.006‐0.011, *P*<.001) and 0.007 (95% CI −0.000 to 0.016, *P*=.06), respectively. Visits beginning with a sub-5-second audio response and ending with text had significantly higher *fv1* than text-only visits, with an AME of 0.069 (95% CI 0.018‐0.120, *P*=.008). The same patterns hold for *fv2* and *fv3*. Based on the Bonferroni test, coefficients with a *P* value smaller than α=.050/3=.017 or α=.50/2=.025 were regarded as significant when evaluating the association with patient loyalty or satisfaction, respectively.

**Conclusions:**

Physician communication practices were significantly associated with patient loyalty and satisfaction. This study is innovative in leveraging large-scale real-world data to systematically examine physician communication. It differs from existing studies by transcending prior survey-based research limitations. It introduces an effective hybrid approach, balancing human connection with text clarity in the field. Its implication in the real world is providing data-driven evidence to guide clinicians and policymakers in designing high-quality telemedicine services.

## Introduction

Telemedicine has emerged as a crucial component of the multichannel service delivery [1-3], significantly expanding access to care beyond the COVID-19 pandemic [4-6]. While granting increased convenience [7,8], enhanced accessibility [9,10], and improved clinical outcomes [11,12], the shift to virtual settings strips away physical interactions, making the communication skills of physicians essential, as they positively influence patient satisfaction and engagement [13-16]. In the specific context of asynchronous telemedicine, where visual cues are lacking, physicians’ choices of text and/or audio messages become critical. Physicians face a dilemma: text messages offer clarity and ease of review, whereas audio communication enables faster expression and conveys a sense of human presence [17-19]. Striking the right balance between these modalities is essential for establishing a robust physician-patient connection [20,21].

Nonetheless, few studies have comprehensively investigated which features of physician response behaviors most effectively enhance the quality of virtual care. Prior research on this topic predominantly focused on in-person visit settings, examining the influence of computer-based electronic medical record writing and patient demographic factors on physician-patient interactions [22,23]. In the context of telemedicine, studies have explored the effect of audio use on satisfaction [24], yielding inconsistent results [18]. Some studies have quantified the length of physician responses and examined its impact on diagnostic accuracy and patient satisfaction using standardized patients [25,26]. Although these studies have value, they exhibit several shortcomings, including the subjectivity of patient satisfaction as an indicator, the artificiality of the standardized patient approach, and constraints on disease categories and sample sizes. Taken together, there is a scarcity of large-scale real-world evidence linking physician communication features to objective behavioral outcomes such as patient loyalty.

The Chinese health care context offers a unique setting to examine patient loyalty due to its high degree of consumer autonomy. Unlike systems with strict gatekeeping, China lacks a mandatory primary care referral system, granting patients direct access to specialists [27-29]. Furthermore, the government-led universal health insurance eliminates restrictive provider networks, allowing patients to seek care from any public institution without facing out-of-network penalties [30]. This autonomy is particularly pronounced in the asynchronous telemedicine examined in this study. In contrast to synchronous virtual visits, which are typically scheduled by physicians for postdischarge or routine follow-up visits based on clinical necessity, asynchronous consultations are patient-initiated and on-demand. Patients voluntarily seek advice, and physicians respond during flexible intervals. Therefore, unconstrained by either insurance policies or provider-mandated appointment requirements, the asynchronous setting effectively isolates patient behavior from structural and clinical constraints. Consequently, within the context of asynchronous consultations in China, patient repurchase acts as a revealed preference, driven predominantly by the overall care experience rather than by insurance constraints or referral mandates.

Our study leverages this unique setting to address the research gap by systematically investigating the association between physician communication features and patient outcomes. The primary objective of this cross-sectional study is to examine how response modality, length, and sequence relate to objective patient loyalty and subjective patient satisfaction. By using large-scale real-world data from 304,337 asynchronous visits at an academic medical center (AMC) in China, we aim to elucidate the distinct utility of these communication features and identify effective engagement patterns. Ultimately, this work is expected to establish an empirical basis for defining quality indicators and improving the patient experience of telemedicine.

## Methods

### Inclusion and Exclusion Criteria

This cross-sectional study was designed and reported in accordance with the Journal Article Reporting Standards (JARS) [31]. Our study used all administrative records (368,317 visits) of telemedicine visits from Peking University Third Hospital, a large AMC in China, covering the period from January 2021 to December 2023. These records were directly exported from the hospital information system (HIS) of the AMC. In this study, a “visit” refers to a complete, billable asynchronous consultation episode, distinct from a single message or reminder. To reflect the dynamics of individual physicians responding to patients’ requests on a daily basis, the following criteria were applied (Multimedia Appendix 1): (1) free telemedicine visits provided by physicians on a voluntary basis were excluded, as physicians’ behavior might change (n=35,804); (2) visits responded to by a medical team, such as multidisciplinary treatments, and those by physicians in diagnostic and therapeutic service departments, were excluded (n=2217); (3) visits initiated by physicians were excluded (n=97); (4) visits where physicians only prescribed medication without replying with a response were excluded (n=7075); (5) to ensure that our results were not skewed by specific physicians, we further excluded visits of “outlier physicians” from the analysis. Specifically, we focused on high-volume physicians, defined as those whose visit counts exceeded the median. We then analyzed the distributions of response volume (number of messages) and duration (length of text/audio). Upon observing structural discontinuities at the extreme tails of these distributions, we applied a gap-based exclusion criterion: the top-ranked physician was removed if their mean value was more than twice that of the second-ranked physician. This conservative threshold was chosen to eliminate extreme anomalies likely driven by nonstandard usage patterns (eg, systematic templates) that could act as high leverage points and skew the regression estimates, while retaining valid high-performing physicians (n=12,086); and (6) given our research interest, we excluded visits containing image responses sent by physicians (n=6701). Altogether, the final dataset contained 304,337 (82.63% of 368,317 typical visits) telemedicine visits provided by 781 unique physicians, requested by 140,466 unique patients.

### Participant Characteristics and Dataset Description

The 781 unique physicians exhibited a balanced gender distribution, with the average age in the mid-forties. Around 85% of individuals were from the fields of internal medicine, surgery, and obstetrics & gynecology. The proportions of different professional titles, such as chief, deputy chief, and attending physicians, were approximately equal (Table 1). Over the 3-year period, the average number of asynchronous visits per patient was 2.069, 1.810, and 1.786 per year. Over 60% of patients attended only once each year (Table 1).

**Table 1.** Baseline characteristics at the visit level (N=304,337), physician level (N=781), and patient level (N=140,466) at Peking University Third Hospital, Beijing, China (2021‐2023).

	2021	2022	2023
Physician level			
Total, n (%)	582 (100)	698 (100)	706 (100)
Field, n (%)			
Obstetrics and gynecology	70 (12)	90 (12.9)	94 (13.3)
Internal medicine	229 (39.3)	280 (40.1)	279 (39.5)
Surgery	185 (31.8)	214 (30.7)	226 (32)
Pediatrics	27 (4.6)	30 (4.3)	28 (4)
Ophthalmology and otorhinolaryngology	71 (12.2)	84 (12)	79 (11.2)
Position at the year, n (%)			
Attending physician	149 (25.6)	224 (32.1)	226 (32)
Deputy chief physician	239 (41.1)	250 (35.8)	252 (35.7)
Chief physician	194 (33.3)	224 (32.1)	228 (32.3)
Gender, n (%)			
Female	303 (52.1)	353 (50.6)	358 (50.7)
Male	279 (47.9)	345 (49.4)	348 (49.3)
Age at the year, mean (SD)	44,735 (8.235)	44,838 (8.381)	45,072 (8.573)
Number of visits per physician, mean (SD)	145,847 (495.547)	145,479 (413.860)	167,011 (503.085)
Patient level			
Total, n (%)	41,033 (100)	56,093 (100)	66,013 (100)
Number of visits, n (%)			
1	24,786 (60.4)	37,733 (67.3)	44,669 (67.7)
2	7268 (17.7)	9011 (16.1)	10,643 (16.1)
3	3416 (8.3)	3815 (6.8)	4443 (6.7)
4	1885 (4.6)	1951 (3.5)	2284 (3.5)
5	1191 (2.9)	1175 (2.1)	1336 (2)
6	757 (1.8)	781 (1.4)	815 (1.2)
≥7	1730 (4.2)	1627 (2.9)	1823 (2.8)
Visit level			
Total, n (%)	84,883 (100)	101,544 (100)	117,910 (100)
Field, n (%)			
Obstetrics and gynecology	47,863 (56.4)	47,980 (47.3)	53,948 (45.8)
Internal medicine	18,622 (21.9)	27,356 (26.9)	28,554 (24.2)
Surgery	13,080 (15.4)	16,733 (16.5)	27,185 (23.1)
Pediatrics	3428 (4)	6192 (6.1)	4417 (3.7)
Ophthalmology and otorhinolaryngology	1890 (2.2)	3283 (3.2)	3806 (3.2)
Position at the year, n (%)			
Attending physician	11,041 (13)	12,685 (12.5)	15,417 (13.1)
Deputy chief physician	26,011 (30.6)	30,704 (30.2)	38,440 (32.6)
Chief physician	47,831 (56.3)	58,155 (57.3)	64,053 (54.3)
Total text length, mean (SD)	70.564 (95.751)	69.445 (92.141)	69.876 (95.651)
Total audio length, mean (SD)	24.261 (41.606)	24.411 (43.024)	22.078 (40.886)

**Figure 1.**
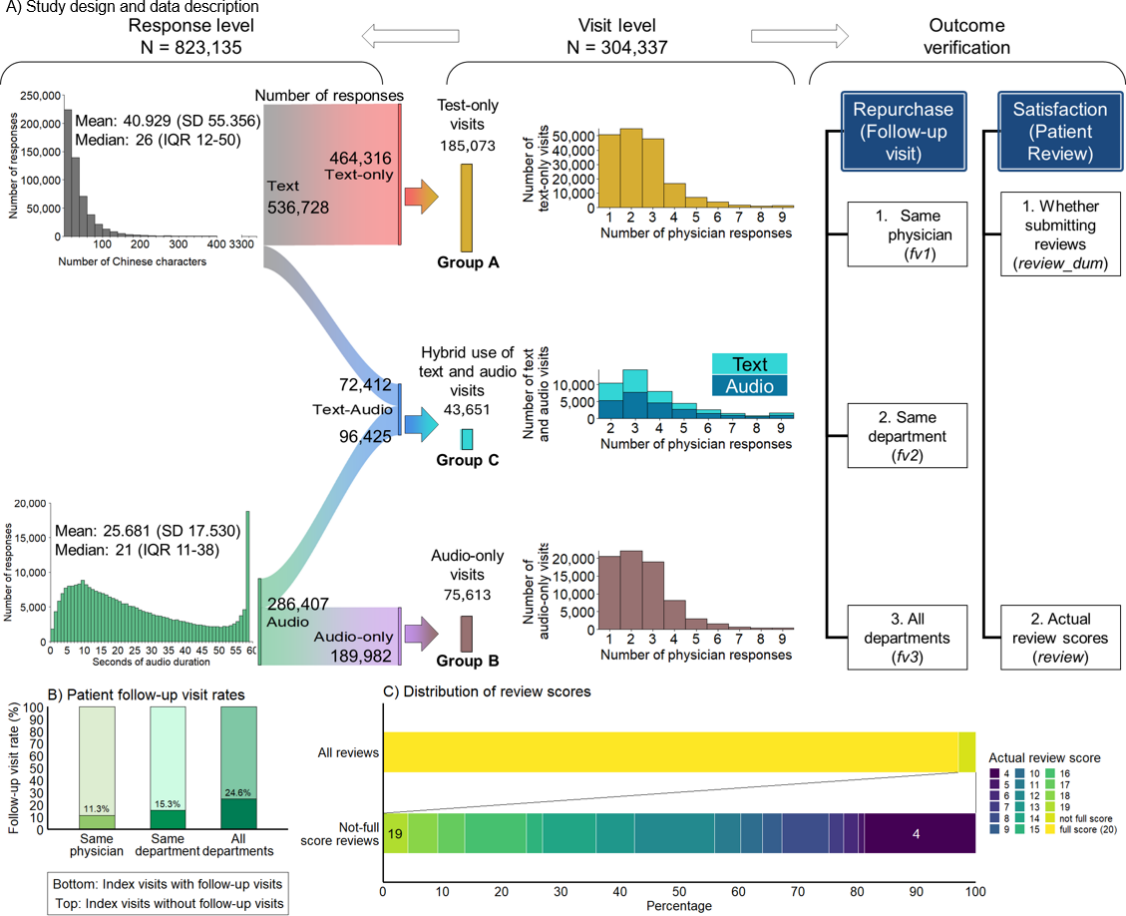
Study overview and distribution of 823,135 physician responses across 304,337 retrospective asynchronous telemedicine visits at Peking University Third Hospital, Beijing, China (2021‐2023).

Around half of the 304,337 visits pertained to obstetrics and gynecology, whereas roughly a quarter were related to internal medicine. The Department of Reproductive Medicine in the field of obstetrics and gynecology had the highest number of asynchronous visits, making up around 40% of the total visits (Figure 1A and Multimedia Appendix 2).

Between 2021 and 2023, the number of visits exhibited a considerable compound annual growth rate of 17.9%. A modest rise in the number of physicians offering asynchronous treatment was observed, with a notable surge in 2022. In 2023, there was a 14.8% (21,533/145,478) rise in the average workload per physician, with significant heterogeneity noticed among individuals.

These 304,337 visits included 823,135 physician responses, translating into 2.705 responses per visit. Of all 823,135 responses, 65.2% (n=536,728) were delivered in text modality and 34.8% (n=286,407) in audio modality. Text responses had no word restriction; however, each audio response was restricted to a maximum duration of 59 seconds, similar to the widely used instant communication application WeChat in China. On average, each text response contained 40.929 Chinese characters, whereas each audio response lasted 25.681 seconds (Figure 1A).

A total of 464,316 text responses formed 185,073 text-only visits, 189,982 audio responses formed 75,613 audio-only visits, and the additional 43,651 visits included both text and audio information. Regardless of the modalities, most visits consisted of no more than 3 physician responses (Figure 1A). Among all index visits, the follow-up visit rates within 6 months were 11.3% (13,937/123,504), 15.3% (15,203/99,126), and 24.8% (19,791/80,557) when considering the scope of the same physician (*fv1*), same department (*fv2*), and all departments (*fv3*) (Figure 1B). Additionally, the percentage of visits reviewed by patients (*review_dum*) was 5% (15,268/304,337), with an average score (*review*) of 19.299 (SD 2.981) out of 20 (Figure 1C).

### Sampling Procedures

This study used a retrospective cross-sectional observational design. All visits that met the predefined eligibility criteria during the study period were included. Therefore, no sampling was performed; instead, the study used a census of all eligible visit records. Accordingly, there were no self-selection concerns in our sample. The dataset was obtained directly from the hospital in Beijing in April 2024 and was analyzed subsequently.

### Sample Size, Power, and Precision

As this was a retrospective study using existing data from HIS, a formal a priori power analysis was not conducted. The sample size was determined by the data in the Peking University Third Hospital within the specified period (ie, January 2021 to December 2023). All eligible records within this timeframe were included to maximize statistical power. Thus, the intended sample size was 368,317 visits. Following the inclusion and exclusion criteria, we achieved an actual sample size of 304,337 visits. The difference resulted from the exclusion process (steps 1‐6 described in Multimedia Appendix 1).

With this substantial sample size, the study is adequately powered to conduct statistical analyses, ensuring high precision in parameter estimates.

### Measures and Covariates

Our study focused on 3 features of physician responses, including the modality of physician responses (text-only, audio-only, or hybrid), response length, and response sequence. Two outcomes (patient loyalty and patient satisfaction) were used to examine the consequences of these physician characteristics.

#### Outcome: Patient Loyalty

Revisit intention is commonly used to measure consumers’ recognition of a product or service and has been studied across various fields, such as tourism, catering, and health care [32-34]. However, revisit intention is a subjective measure that may lack accuracy. Thus, we focused on actual revisits, which provide an objective description of behavior. To distinguish behavioral loyalty from clinical dependency and avoid confusion with the negative quality indicator “revisit,” we specifically used “follow-up visit” as a proxy for patient loyalty.

This measurement strategy is grounded in 2 key premises. First, revisits within the acute phase (eg, 14, 21, or 30 d) often serve as negative indicators in health care, reflecting unresolved medical issues or unmet expectations requiring additional treatment in a relatively short period of time [35-41]. Second, in this setting where patients have sufficient autonomy to choose providers for ongoing care, a voluntary return after the acute phase reflects a revealed preference rather than a lack of alternatives [42]. In this context, a follow-up visit is defined as the patient reusing the asynchronous service after a specific exclusion period following the index visit. Based on this rationale, a subsample of index visits was constructed to accurately capture this long-term behavioral loyalty.

**Figure 2.**
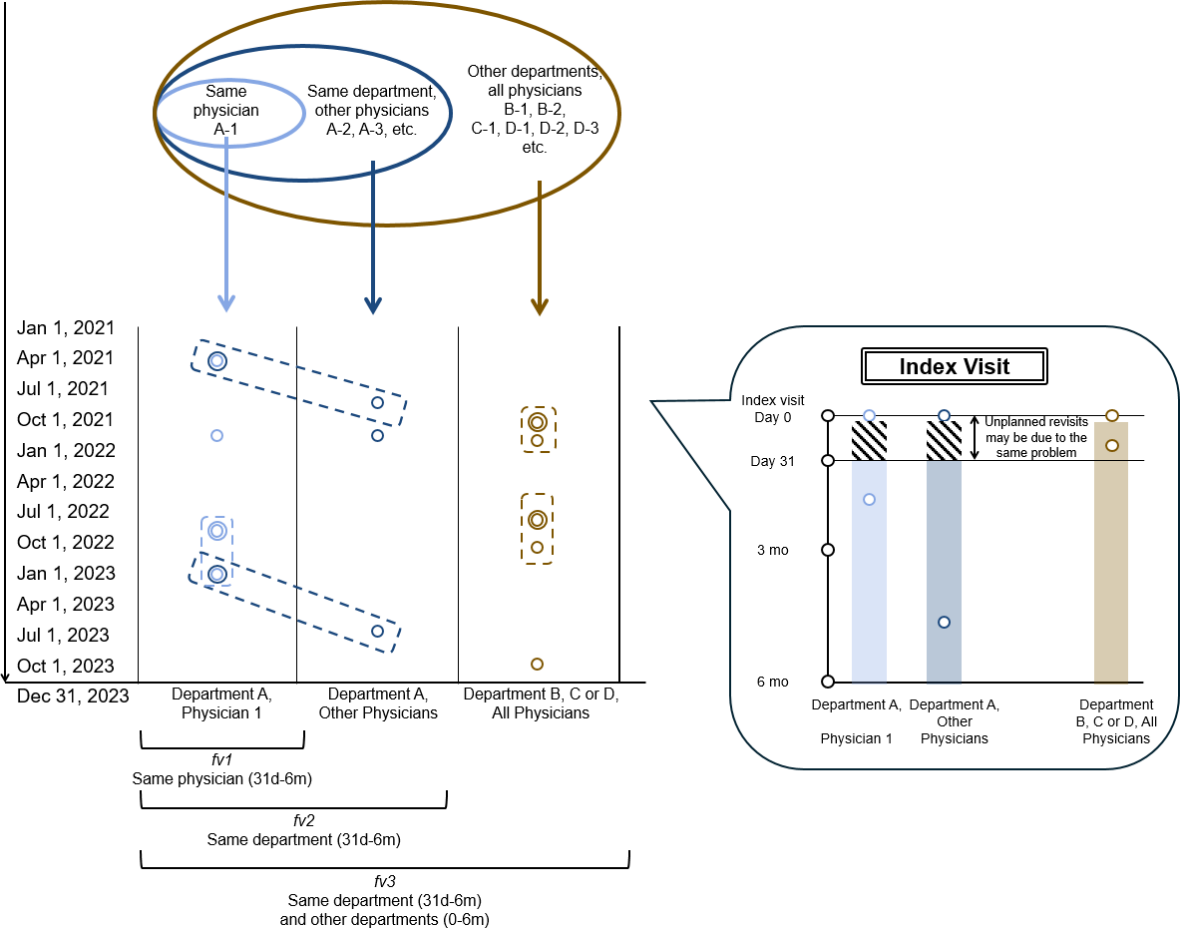
Schematic illustration of index and follow-up visits used to measure patient loyalty across 3 hierarchical scopes: same physician (*fv1*, light blue circle), same department (*fv2*, dark blue circle), and same hospital (*fv3*, gold circle).

To do so, we defined 2 dimensions—period and scope—to help formulate the concept of an index visit and a follow-up visit. The period was considered to be 6 months (182 d), which was the typical timeframe that the Clinician & Group Survey used to assess the lasting effects of a visit [43,44]. Meanwhile, the scope varied across different measurements of visit loyalty. Specifically, the scopes of (1) the same physician, (2) the same department, and (3) all departments applied to the 3 measures: follow-up visit 1 (*fv1*), follow-up visit 2 (*fv2*), and follow-up visit 3 (*fv3*), respectively. The 3 subsamples of index visits were thus defined as the visits without any visit with the same physician, in the same department, or in all departments in the 6 months preceding them (Figure 2).

If there was at least one follow-up visit with the same physician, within the same department, or across all departments within 6 months after the corresponding index visit, then *fv1*, *fv2*, or *fv3* equaled 1; otherwise, it was equal to 0. Meanwhile, index visits followed by a visit to the same physician within the subsequent 30 days were excluded for *fv1*, whereas those followed by a visit within the same department were excluded for *fv2* and *fv3*, as immediate follow-up visits were often regarded as a signal of poor quality [35-41]. Note that when the scope was extended to all departments (ie, *fv3*), only follow-up visits within the same department within 30 days were excluded, whereas cross-department visits were retained, as these cross-department follow-up visits were unlikely to reflect poor quality of care and were more likely to indicate new demand. Finally, to ensure we could observe the entire 6-month period before and after the index visits, we excluded 2 types of index visits for all 3 measurements: (1) those in the last 6 months of our sample period and (2) those in the first 6 months of our sample period. In this way, we obtained 123,504, 99,126, and 80,556 index visits for *fv1*, *fv2*, and *fv3*, respectively.

#### Outcome: Patient Satisfaction

Patient satisfaction is commonly used as an evaluation metric for services in health care settings [45]. Listed as 1 of the 5 domains of the Quintuple Aim, patient satisfaction is strongly related to quality [46-48]. Furthermore, research has demonstrated that it is positively associated with patients’ intention to revisit [34,45,49].

In our study, patients could review their experience on a voluntary basis after having asynchronous visits. The review asked the patients to rate their experience on a scale ranging from a minimum of 4 points to a maximum of 20 points. Based on this, 2 measurements were constructed: a dummy variable capturing whether the patient submitted a review (*review_dum*) and the corresponding review score conditional on the submission (*review*).

#### Control Variables

We defined some other variables to describe the characteristics of physicians and added them as control variables in the regression analyses. Based on the classification of departments in the sample hospital and different disease types, all departments were divided into internal medicine, surgery, obstetrics and gynecology, pediatrics, and ophthalmology and otorhinolaryngology (including ophthalmology, otolaryngology [ENT], and stomatology). Each field was defined as a dummy variable. Furthermore, 3 dummy variables were defined for attending physicians, deputy chief physicians, and chief physicians, respectively. Additionally, we counted the number of Chinese characters and the seconds of audio duration by physicians per visit as control variables.

### Data Collection

Data were directly extracted from the HIS of Peking University Third Hospital. The dataset integrates administrative records (eg, timestamps and payment status) and clinical contents (eg, message modality and response length).

### Quality of Measurements

We used all data exactly as recorded in the system. This direct-output process did not involve human data collectors, thus eliminating any potential bias or intercollector variability associated with manual data collection.

### Instrumentation

In this study, we developed a set of instruments to comprehensively assess patient loyalty and satisfaction. Loyalty was evaluated through follow-up visits, defined within different periods and scopes. Satisfaction was measured based on the raw review score provided by the patient, including whether a review was submitted and the review score itself.

### Masking

Because this was a retrospective study rather than a randomized controlled trial, blinding was not applicable, as data collection occurred after clinical interactions had been completed.

### Conditions and Design

This study used a nonexperimental design (ie, no experimental manipulation). As an observational design, it used retrospective data.

### Data Diagnostics

There were no missing data in the dataset, ensuring a complete set of records for analysis. All visit-level continuous variables were censored at the top 1% to alleviate concerns related to outliers.

### Analytic Strategy

All statistical analyses and visualizations were performed using Stata (MP 18.0), R (R/2024.04.2+764), and Origin (2024bSr0H).

To examine the role of the response modality, we split the whole sample into 3 subsamples: text-only visits (Group A), audio-only visits (Group B), and visits with both modalities (Group C) (Figure 1A). We also created 3 dummy variables to indicate each subsample, respectively. In the regression, we included 2 dummies for Groups B and C, as well as control variables for physician title (a dummy for deputy chief physicians and another for chief physicians). Moreover, the day-of-week fixed effect was added to capture features specific to the day of the week, such as weekdays and weekends. The year-month fixed effect was added to capture month-specific shocks common to all physicians, such as seasonal fluctuations in workload. The physician fixed effect was also included to capture individual behavioral habits. Standard errors were clustered at the physician level. Probit regressions were applied when the dependent variables were *fv1*, *fv2*, *fv3*, and *review_dum* using the Stata command *probit*, and the average marginal effects (AMEs) were reported. Ordinary least squares regressions were applied when the dependent variable was *review* using the Stata command *reghdfe*. Notably, only a subsample of visits contained reviews, which might lead to sample selection concerns. To account for such bias, we followed the standard procedure of the Heckman two-step method [50] by calculating the inverse Mills ratio and adding it into the regression in the second step. To further examine the differences between visits with both text and audio responses and those with only audio responses, we used the Wald test with the Stata command *test* after the regression to examine whether the coefficients of being in Group B and Group C were equivalent.

In a similar vein, we conducted regression analysis using the same models and dependent variables to evaluate the extent to which the length of responses affects patient loyalty and patient satisfaction. Specifically, we included the number of text responses (*text_num*) and the number of audio responses (*audio_num*) as the main independent variables and controlled for the total Chinese characters and total audio duration. Additionally, we added the same set of control variables and fixed effects as in the analysis regarding the response modality. Moreover, as a companion exercise, we further considered the extreme cases of visits with only audio responses, where the total audio duration falls within the range of 55‐59 seconds (Group B1). We investigated whether splitting long audio responses mattered by including *audio_num* while controlling for the total audio duration.

We next considered the role of the sequence of audio responses. Specifically, we constructed a subsample that included 2 types of visits, both of which included only one audio response. The first started with the audio response (Group C1), whereas the second ended with the audio response (Group C2). We constructed a dummy variable to indicate the second type of visit. By incorporating the dummy variable, as well as controlling for *text_num*, *audio_num*, total Chinese characters, total audio duration, the physician title, and 3 sets of fixed effects, the coefficient of the dummy represented the difference in patient loyalty and satisfaction associated with moving the audio response from the opening to the ending.

The 3 sets of examinations corresponded to our prespecified hypotheses regarding response modalities, response length, and response sequence, each comprising 5 tests. To control the family-wise Type I error rate arising from multiple comparisons, Bonferroni correction was applied. The 3 outcomes related to patient loyalty (*fv1*, *fv2*, and *fv3*) and the 2 outcomes related to patient satisfaction (whether a review was provided and the review score) were each treated as a separate family. Accordingly, *P* values smaller than α=.050/3=.017 for loyalty outcomes and α=.050/2=.025 for satisfaction outcomes were considered statistically significant under the Bonferroni correction.

Then, as an exploratory analysis, the length and position of audio responses were jointly considered using additionally constructed subsamples that also contained 2 types of visits. The first type required the visit to start with the audio response, while the length was required to be shorter than the threshold *t*, ranging from 2 to 60 seconds. The second type included all text-only visits (Group A). After including the dummy variable for Group A as an independent variable, we repeated the above regression analysis using different subsamples with different thresholds *t* combined with Group A, thus obtaining 59 regression results.

Beyond the dimension of audio length, the variation in position was also considered. We constructed a subsample containing visits with one audio response shorter than 5 seconds while not in the first position, as well as text-only visits (Group A). All visits were required to have at least 2 physician responses to facilitate the comparison. Using the subsample and constructing a dummy for the visits with audio responses, we could examine the difference between the above 2 types of visits regarding outcomes.

### Ethical Considerations

This study protocol was approved by the Institutional Review Board (IRB) of Peking University Third Hospital (approval number IRB00006761-M2024701). The requirement for informed consent was waived by the IRB due to the retrospective nature of the study and the use of deidentified data. All data were strictly anonymized and deidentified prior to analysis to protect participant privacy and confidentiality. No compensation was provided to participants for this study. No identifiable images or personal details of participants were included in this manuscript.

## Results

### Response Modalities

**Figure 3.**
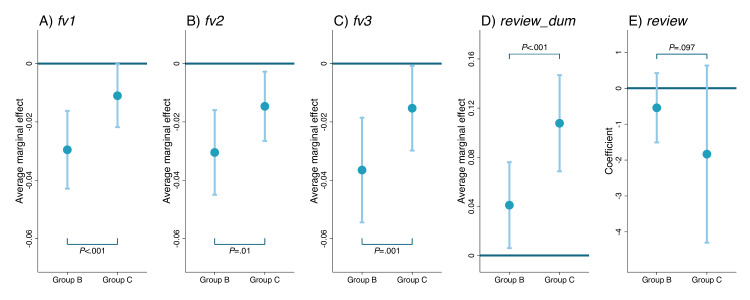
Association between response modality and outcomes (N=304,337 visits) at Peking University Third Hospital, Beijing, China (2021‐2023). Each subfigure reports the results from one regression. The blue dots represent average marginal effects on *fv1*, *fv2*, *fv3*, and *review_dum* in Probit regressions (A-D) and coefficients for *review* in the ordinary least squares regression (E). Light blue vertical lines with caps represent 95% CIs. Wald tests were used to examine whether the coefficients of the 2 dummies were equal.

As for the follow-up visit rates, the AME for pure audio response visits was −0.030 (95% CI −0.043 to −0.016, *P*<.001), −0.030 (95% CI −0.045 to −0.016, *P*<.001), and −0.037 (95% CI −0.054 to −0.019, *P*<.001) for *fv1*, *fv2*, and *fv3*, respectively, indicating that the follow-up rates for pure audio visits were 3‐4 percentage points lower than those for pure text visits (Figure 3). While the combination of text and audio surpassed pure audio in eliciting follow-up visits, its coefficients remained negative in comparison to pure text. In summary, text-only visits (Group A) were associated with the highest patient loyalty, followed by combinations of text and audio (Group C), while audio-only visits (Group B) performed the worst, obtaining 16.6%‐30.9% lower follow-up visit rates than those achieved by text-only visits.

As for the review, whether the use of audio would promote the likelihood of receiving reviews was examined first. The AME of pure audio visits was 0.041 (95% CI 0.006‐0.076, *P*=.02), indicating that physicians were more likely to receive patient reviews following the provision of medical services in the pure audio modality. The AME for the combination of text and audio was 0.108 (95% CI 0.068‐0.147, *P*<.001), suggesting that visits in mixed information modalities were most evaluated by patients, followed by those in pure audio, while those in pure text were the least. Subsequently, the linkage between various response modalities and review scores was investigated. Upon adjusting for the inverse Mills ratio, it was determined that there were no statistically significant differences in the review scores among Groups A, B, and C. Note that Group B could achieve a higher level of satisfaction than Group A when the inverse Mills ratio was not controlled (Multimedia Appendix 3).

### Response Length

Alongside the modality of response, length is a critical measure in telemedicine. Therefore, the association between response length and outcomes was further investigated. The number of responses was used as a metric to measure visit length when the total information load was fixed.

**Figure 4.**
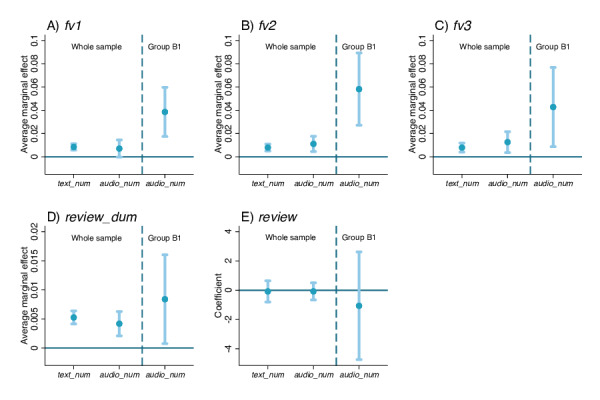
Association between response length and outcomes at Peking University Third Hospital, Beijing, China (2021‐2023). Each subfigure reports the results of 2 regressions, using the whole sample (N=304,337 visits) and Group B1 (n=3674 visits), respectively. Blue dots represent average marginal effects on *fv1*, *fv2*, *fv3*, and *review_dum* in probit regressions (A-D) and coefficients for *review* in ordinary least squares regressions (E). Light blue vertical lines with caps represent 95% CIs.

As for patient loyalty, results demonstrated that the AMEs of *text_num* were 0.009 (95% CI 0.006‐0.011, *P*<.001), 0.008 (95% CI 0.005‐0.011, *P*<.001), and 0.008 (95% CI 0.004−0.012 *P*<.001) for *fv1*, *fv2*, and *fv3,* respectively. The AMEs of *audio_num* were 0.007 (95% CI −0.000 to 0.016, *P*=.06), 0.011 (95% CI 0.005−0.018, *P*<.001), and 0.013 (95% CI 0.004‐0.022, *P*=.006), respectively (whole sample, Figure 4). The findings indicated that irrespective of the modality—text or audio—used for telemedicine services, when the information load was fixed, an increased number of physician responses correlated with enhanced patient loyalty.

To further validate the above conclusions, we leveraged the design of the telemedicine platform, where a single audio response was limited to a maximum of 59 seconds, to create a more extreme scenario. Specifically, cases where the total audio length of physician responses in a visit fell between 55 and 59 seconds were selected, and we investigated whether there would be differences in the outcome measures as the number of audio responses increased (Group B1, Figure 4).

The results showed that the AMEs of *audio_num* were 0.039 (95% CI 0.017‐0.060, *P*<.001), 0.058 (95% CI 0.027‐0.089, *P*<.001), and 0.043 (95% CI 0.009‐0.077, *P*=.014) for *fv1*, *fv2*, and *fv3*, respectively (Figure 4). This positive association further confirmed that patient loyalty improved when lengthy audio information was divided into several shorter segments.

As for the review, each additional text and audio response was linked to a 0.5% (95% CI 0.4%‐0.6%, *P*<.001) and 0.4% (95% CI 0.2%‐0.6%, *P*<.001) increase in the probabilities of patients providing reviews, respectively. Yet, both additional text and audio responses did not significantly alter the actual review score, indicating that segmenting text or audio into shorter responses was not associated with improved patient satisfaction (Figure 4).

### Sequence

In addition to response length, the sequence in which text and audio messages were presented may also be connected with outcomes in visits containing both modalities. We restricted the sample to Group C to examine the association of audio responses positioned at various rounds and the outcomes. Specifically, 2 groups were constructed from the sample. The first group comprised visits in which the first response was in the audio modality and the last response was not (audio-opening visits, Group C1). The second group encompassed visits that ended with an audio response and did not begin with an audio response (audio-ending visits, Group C2).

Firstly, the follow-up visit rates associated with these 2 groups of visits were evaluated. The findings indicated that the AMEs for being in Group C2 were −0.019 (95% CI −0.033 to −0.005, *P*=.007), −0.022 (95% CI −0.042 to −0.001, *P*=.04), and −0.023 (95% CI −0.045 to −0.000, *P*=.046) on *fv1*, *fv2*, and *fv3*, respectively, compared to Group C1 (Table 2). This demonstrated that patients exhibited increased loyalty to the same physician when physicians used audio responses at the beginning of a telemedicine session instead of at the end. Note that the associations for *fv2* and *fv3* were no longer significant after Bonferroni correction.

**Table 2.** Association between the position and sequence of the audio responses and outcomes at Peking University Third Hospital, Beijing, China (2021‐2023).^a^

	*fv1*	*fv2*	*fv3*	*review_dum*	*review*
Comparing the audio-opening visits (Group C1) and audio-ending visits (Group C2)					
Group C2 (95% CI, *P* value)	−0.019 (−0.033 to −0.005, *P*=.007)	−0.022 (−0.042 to −0.001, *P*=.04)	−0.023 (−0.045 to −0.000, *P*=.046)	0.005 (−0.001 to 0.011, *P*=.098)	0.170 (−1.017 to 1.358, *P=*.78)
Comparing the visits with nonbeginning short audio group and text-only visits (Group A)					
Visits with nonbeginning short audio (95% CI, *P* value)	0.152 (−0.032 to 0.337, *P*=.11)	0.189 (−0.045 to 0.422, *P*=.11)	0.212 (−0.150 to 0.575, *P*=.25)	−0.055 (−0.188 to 0.077, *P*=.41)	−1.960 (−15.598 to 11.677, *P*=.78)
Controls	Y	Y	Y	Y	Y
Physician FE^b^	Y	Y	Y	Y	Y
Day-of-week FE	Y	Y	Y	Y	Y
Year-month FE	Y	Y	Y	Y	Y

a The coefficients in the first row were estimated using the subsample Group C1 and Group C2 (n=33,758 visits), and those in the second row were estimated using the subsample Group A and visits with only one nonbeginning short audio response of less than 5 seconds in Group C, both restricted to visits with at least 2 physician responses (n=134,698 visits). Average marginal effects for *fv1*, *fv2*, *fv3*, and *review_dum* from probit regressions are reported in the first 4 columns, and coefficients for *review* from ordinary least squares regressions are reported in the last column.

b FE: fixed effect ____.

Secondly, the association between audio position and reviews was examined. The findings indicated no significant association between audio position and the probability of patients submitting reviews or the actual review scores.

### Role of Audio

The previous results suggested that, while the audio modality did not facilitate the follow-up visit rate, as reflected by a diminished patient loyalty for Group C compared to Group A, brevity and being in the opening position of audio responses were individually associated with elevated follow-up visit rates. Therefore, the follow-up visit rates of Group C1 and Group A were compared to examine if the decrease in follow-up visit rates due to audio use could be mitigated when the audio was short and positioned at the beginning.

**Figure 5.**
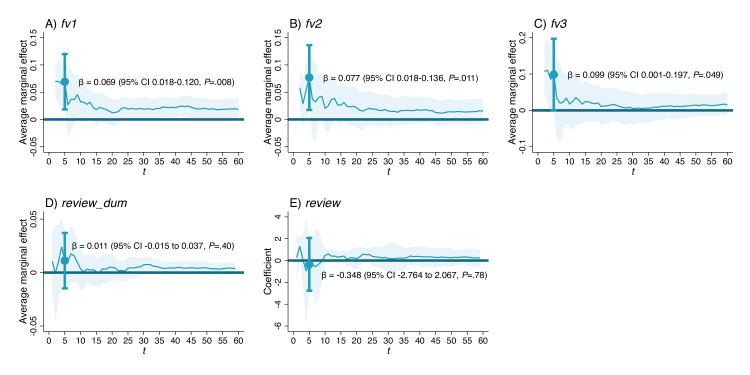
Association between the length of the first audio response and outcomes at Peking University Third Hospital, Beijing, China (2021‐2023). Each subfigure contains results from 59 regressions, each comparing visits with only 1 audio response placed at the first position and shorter than *t* seconds (2≤t≤60) with visits consisting solely of text. The blue curves represent average marginal effect (AMEs) of being the visit with an introductory audio response on *fv1*, *fv2*, *fv3,* and *review_dum* in probit regressions in (A-D), and its coefficients on *review* in ordinary least squares regressions (E). The light blue shades represent 95% CIs. The blue dots and vertical lines with caps represent the AMEs or coefficient and 95% CIs, respectively, when *t*=5 (meaning subsample with pure-text visits and visits with an introductory audio response that are shorter than 5 s).

Results showed that when the length of the introductory audio was less than 5 seconds, the follow-up visit rates in this group were significantly higher than those of visits with only text, with the AMEs of 0.069 (95% CI 0.018‐0.120, *P*=.008), 0.077 (95% CI 0.018‐0.136, *P*=.011), and 0.099 (95% CI 0.001‐0.197, *P*=.049) on *fv1*, *fv2*, and *fv3*, respectively (Figure 5). As for reviews, no matter how the length of the introductory audio response changed, there were no significant differences between Group C1 and the text-only visits in terms of the probability of getting reviews or the actual review scores.

Therefore, visits featuring solely brief introductory audio responses yielded a higher follow-up visit rate than those with only text responses. In addition, this effect diminished when the audio length reached 5 seconds.

Subsequently, short duration (less than 5 s) was maintained as a favorable condition, and whether changing the position of short audio responses would be associated with elevated or diminished follow-up visit rates compared to Group A was assessed. We identified visits in Group C that contained only one short audio response, which was not positioned in the first place, as the nonbeginning audio response group, then compared this group to text-only visits with at least 2 physician responses. No significant effects were observed between the 2 groups of visits (Table 2).

## Discussion

### Principal Results

This study is the first to systematically examine the effects of physicians’ communication modality, length, and sequence on patient behavioral outcomes in asynchronous telemedicine. Our primary findings indicated that the repurchase rate was highest in text-only visits, followed by text-audio hybrid visits, and lowest in audio-only visits. The reduced average length of both text and audio responses was correlated with higher patient loyalty. We also identified a response pattern in which virtual visits begin with a brief introductory audio message followed by text, which was associated with the highest patient loyalty. Additionally, audio responses were connected to a higher likelihood of patients providing reviews without changing the review scores. Our research illustrates specific behavioral patterns of physicians in telemedicine, elucidates the association between various communication features and outcomes, and serves as a reference for delivering high-quality and efficient telemedicine services.

This study improves our current comprehension of physician behaviors in telemedicine by leveraging a large-scale dataset of real-world interactions. A key methodological strength lies in our dual-outcome measurement, which integrates objective longitudinal repurchase behavior with subjective satisfaction scores. While satisfaction may accurately represent the immediate consumption experience, it remains a subjective assessment prone to selection bias, often suffering from a low response rate, which is estimated to be only 5%‐10% in natural settings [51]. In contrast, repurchase behavior serves as an objective, long-term metric free from selection bias [52,53]. By combining these 2 metrics, our study overcomes the limitations of relying solely on self-reported data [34,54,55], offering a more comprehensive evaluation of patient outcomes. In addition, our study thoroughly characterized various dimensions of physician responses, including the modality, length, and sequence. This multidimensional analysis approach establishes a robust empirical foundation for understanding physician-patient communication behaviors.

Central to the validity of this multidimensional framework is the rigorous construction process of the patient loyalty proxy, which was carefully designed to distinguish behavioral preference from clinical necessity or dependency. Unlike in general consumer services, a repeat visit in health care does not inherently signal satisfaction; it may instead reflect unresolved medical needs [56,57]. To address this problem, we imposed specific temporal and scope constraints to isolate true loyalty.

Regarding the temporal dimension, we established a 1‐ to 6-month window to balance the exclusion of clinical necessity against the attribution of care quality. On the lower bound, we excluded revisits occurring within 30 days [38]. In clinical contexts, immediate returns often serve as proxies for unplanned revisits, signaling ineffective initial communication or unresolved symptoms rather than satisfaction [35-41]. On the upper bound, we restricted the observation to 6 months. This cutoff is empirically grounded in the temporal decay of visit influence; for instance, prior research indicates that while initial patient experience significantly predicts retention at 6 months, this association fades and becomes nonsignificant by 12 months [43,44]. Thus, limiting the window ensures that the measured follow-up behavior remains attributable to the impact of the index visit rather than long-term habituation.

Regarding the scope of loyalty, we stratified follow-up visits into 3 levels—the same physician, the same department, and all departments—to capture the multilayered nature of patient trust [58]. While returning to the same physician (*fv1*) serves as the most direct measure of interpersonal loyalty, patients in high-demand academic centers often manifest institutional trust by transferring their confidence to a physician’s colleagues [59,60]. By broadening the scope to the same department (*fv2*) and all departments (*fv3*), we capture the spillover effect of effective communication, where a positive interaction with an individual provider enhances the perceived reliability of the entire medical team [59,60].

Crucially, our exclusion criteria incorporated a nuanced interaction between time and scope to account for disease relatedness. We applied the 30-day exclusion period strictly to revisits involving the same physician or department, as these were highly likely to address the same unresolved medical needs. In contrast, visits to different departments within a month were not subject to this exclusion constraint. This distinction is pivotal, as an immediate return to the same specialty suggests a failure to resolve the initial problem, whereas a visit to a different specialty typically indicates a new, unrelated health need [61]. Thus, retaining these cross-specialty visits allows us to capture a broader form of hospital-wide loyalty without confounding it with the negative quality signal of clinical redundancy. Yet, given that returning to the same individual physician offers the most direct and unmediated reflection of the specific interaction’s quality, we designated *fv1* as our primary outcome measure to maximize internal validity.

Our focus on repurchase behavior complements the broader literature on patient nonattendance [16]. While nonattendance metrics capture passive adherence to scheduled appointments, the asynchronous workflow in our study operates on a patient-initiated, on-demand basis where prebooking is absent [62]. In this context, engagement is not measured by the absence of a no-show but by the active decision to repurchase. Thus, our loyalty metric represents a deliberate reselection of the provider, serving as a distinct, proactive indicator of the patient-provider connection.

The specific aspects that these 2 outcome indicators highlight align with the different factors that patients encounter during a telemedicine visit. In a virtual visit, a patient’s holistic experience is derived from 2 distinct aspects: the comprehension of medical information, which constitutes an evaluation of factual matters, and the perception of physicians’ attitudes, which represents an evaluation of interpersonal interactions. We shall examine the importance of the findings from these 2 aspects [63].

Research indicated that comprehension of information was a significant determinant in fostering repurchase behavior [64]. Textual information surpasses auditory in improving understanding of lengthy and complicated material [65]. Meanwhile, medicine is considered a complex field that most individuals have limited knowledge of, resulting in significant information asymmetry [66]. These could explain our findings that text-only visits resulted in more repurchasing behaviors. Because text responses may improve patients’ comprehension of physicians’ information and provide convenient access for future reference, thereby fostering recognition and promoting repurchases, the outcome metric from an objective and long-term perspective.

Another crucial dimension of outcomes is patient satisfaction, which primarily reflects patients’ feelings and attitudes toward physicians. Prior research has indicated that audio communication is more effective than text in bridging gaps among those unable to meet in person [42], and it correlated with increased satisfaction [24]. However, in this study, the use of audio responses resulted in an enhancement of the probability of providing reviews, rather than review scores after adjusting for the inverse Mills ratio. Yet, in the absence of controlling for selection bias, the review scores were higher in the audio-only visits (*P*=.04), leading us to hypothesize that the increase in satisfaction levels attributed to audio might be a result of selection bias.

Beyond the response modality, a notable discovery is that shorter responses had better outcomes not only for audio responses but also for text responses. When the load of information from the physician was consistent, dividing the content into shorter responses correlated with a higher probability of follow-up visits and an enhanced inclination to provide feedback. This finding aligns with previous empirical research conducted in online health communities, which demonstrated that although physicians’ knowledge-sharing behaviors could effectively enhance patient engagement, overly lengthy medical information might inadvertently induce patient anxiety [67], suggesting the importance of response segmentation in physician-patient communication.

Our findings indicated that audio responses were adversely associated with patients’ repurchase behavior. The repurchase rate for audio-only or hybrid visits was inferior to that of text alone. Does this imply that audio should not be used at all? Not particularly. We identified that visits featuring a brief introductory audio message (<5 s) followed by text yielded the highest patient loyalty, highlighting it as a particularly effective communication pattern within our sample. This served as evidence that audio itself was not inherently flawed. Given the inability to access substantive content based on communication norms, we hypothesize that these initial, sub–5-second audio clips likely function as personalized greetings or empathetic openings rather than complex medical explanations. This initial human touch may help establish an emotional connection, while the subsequent text ensures the clarity and reviewability of the medical advice.

### Limitations

This study used physician-patient interaction records in the real world and thoroughly examined the roles of text and audio usage, offering a reference for physicians in telemedicine practice; nevertheless, it also had specific limitations.

First, this study focused on observable communication features and did not examine the substantive content of asynchronous care. As a result, the informational richness and clinical appropriateness of medical advice, as well as their roles in shaping communication practices, could not be directly assessed. Future research may extend this work by incorporating content-based or semantic analysis with data on medical specialization and case characteristics to provide a more comprehensive evaluation of how communication quality and clinical complexity jointly affect telemedicine outcomes.

Second, while our study documents associations between physicians’ response features and patient behavioral outcomes, it does not allow us to infer causal relationships. Retrospective investigations do indeed possess such limitations. To alleviate the concern, we prioritized controlling for a series of covariates and added abundant fixed effects at the physician level, day-of-week level, and year-month level. Therefore, our results are unlikely to be driven by differences across physicians or time periods. While we cannot fully eliminate endogeneity in a retrospective study, we have taken extensive steps to mitigate such concerns in our analysis.

Third, the external validity of our findings may be influenced by institutional, platform, and cultural factors. The data were drawn from a single large AMC in China, where high patient volume and strong professional norms may shape communication patterns differently from those in primary or community care settings. In addition, platform-specific design features, such as the 59-second audio length limit, may act as technological nudges encouraging concise responses, which is a common constraint across major Chinese platforms. These factors could limit generalizability to other health care systems or international contexts with different technical parameters and cultural expectations around physician-patient interactions. Future cross-institutional, cross-platform, and cross-cultural studies are needed to examine how these elements interact to shape effective communication practices.

### Conclusions

This study is innovative in using a large-scale dataset to thoroughly examine the distinct links between specific communication features and patient loyalty outcomes in telemedicine. It differs from existing studies by leveraging a large-scale real-world dataset and objective indicators, diverging from prior research that relied on small-scale simulations or subjective surveys. It provides critical empirical evidence to the field, establishing that while text-only visits facilitate content comprehension and audio encourages feedback, a hybrid pattern with a brief audio introduction followed by text is associated with the highest patient loyalty. Its implication in the real world is offering critical knowledge on how physicians can strategically select engagement tactics to improve service quality and patient retention.

## Supplementary material

10.2196/86977 Multimedia Appendix 1 Flowchart of virtual visits after applying exclusion and inclusion criteria.

10.2196/86977 Multimedia Appendix 2 The number of visits (N=304,337 visits) and physicians (N=781) across years and departments at Peking University Third Hospital, Beijing, China (2021-2023). Departments with visit volume of less than 1% each year are consolidated into “Others.”

10.2196/86977 Multimedia Appendix 3 The impact of response modality on satisfaction without controlling inverse Mills ratio (N=304,337 visits) at Peking University Third Hospital, Beijing, China (2021-2023).

## Abbreviations

AMC: academic medical center
AME: average marginal effect
HIS: hospital information system
IRB: Institutional Review Board
JARS: Journal Article Reporting Standards
