# Supplementary figures and images for: Association Between Physician Communication Features and Patient Outcomes in Telemedicine: Retrospective Cross-Sectional Observational Study
Source: J Med Internet Res. 2026 Mar 26;28:e86977. doi: 10.2196/86977 (PMC13021109; doi:10.2196/86977)

Flowchart of virtual visits after applying exclusion and inclusion criteria


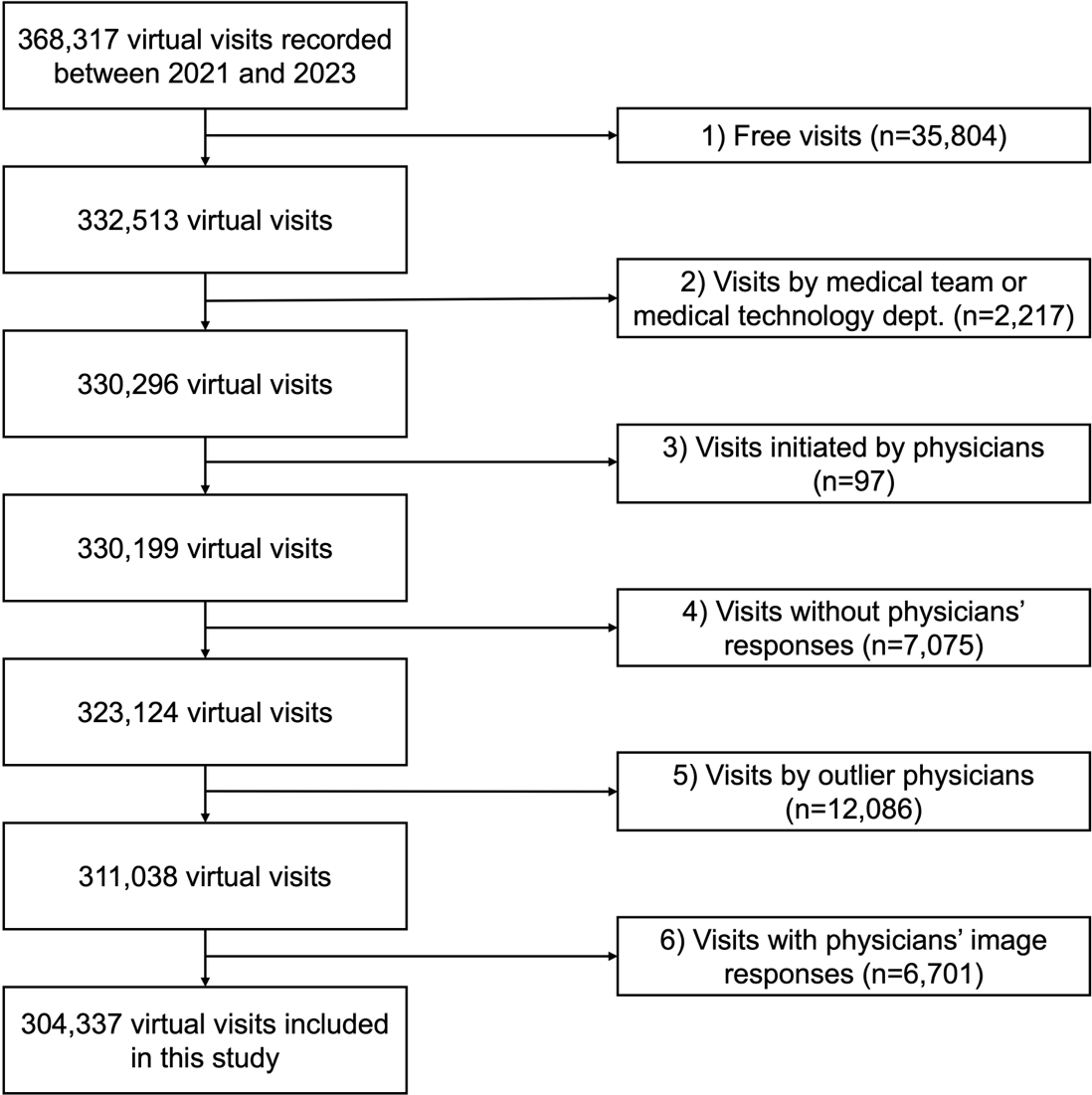

Supplement: Multimedia Appendix 1 [file jmir-v28-e86977-s001.docx]
