# Supplementary material for: Association Between Physician Communication Features and Patient Outcomes in Telemedicine: Retrospective Cross-Sectional Observational Study
Source: J Med Internet Res. 2026 Mar 26;28:e86977. doi: 10.2196/86977 (PMC13021109; doi:10.2196/86977)
Supplement: Multimedia Appendix 2 [file jmir-v28-e86977-s002.docx]

The number of visits (N=304,337 visits) and physicians (N=781) across years and departments at Peking University Third Hospital, Beijing, China (2021–2023). Departments with visit volume of less than 1% each year are consolidated into “Others”.

| Field | Department, n (%) | Number of Visits | | | | Number of Physicians | | |
| --- | --- | --- | --- | --- | --- | --- | --- | --- |
|  |  | 2021 | 2022 | 2023 | Total | 2021 | 2022 | 2023 |
| Obstetrics & Gynaecology | Reproductive Medicine | 39,939 (47.1) | 36,931 (36.4) | 40,865 (34.7) | 117,735 (38.7) | 36 (6.2) | 41 (5.9) | 46 (6.5) |
|  | Obstetrics | 3,612 (4.3) | 6,223 (6.1) | 7,340 (6.2) | 17,175 (5.6) | 13 (2.2) | 20 (2.9) | 18 (2.5) |
|  | Gynaecology | 4,312 (5.1) | 4,826 (4.8) | 5,743 (4.9) | 14,881 (4.9) | 21 (3.6) | 29 (4.2) | 30 (4.2) |
| Internal Medicine | Dermatology | 2,211 (2.6) | 3,470 (3.4) | 5,855 (5.0) | 11,536 (3.8) | 19 (3.3) | 19 (2.7) | 20 (2.8) |
|  | Endocrinology | 2,666 (3.1) | 3,856 (3.8) | 4,751 (4.0) | 11,273 (3.7) | 12 (2.1) | 15 (2.1) | 15 (2.1) |
|  | Respiratory Medicine | 415 (0.5) | 5,808 (5.7) | 2,836 (2.4) | 9,059 (3.0) | 20 (3.4) | 26 (3.7) | 22 (3.1) |
|  | Gastroenterology | 2,041 (2.4) | 2,508 (2.5) | 2,835 (2.4) | 7,384 (2.4) | 24 (4.1) | 26 (3.7) | 27 (3.8) |
|  | Rheumatology and Immunology | 2,750 (3.2) | 1,887 (1.9) | 1,942 (1.6) | 6,579 (2.2) | 12 (2.1) | 11 (1.6) | 10 (1.4) |
|  | Medical Oncology and Radiation Sickness | 4,592 (5.4) | 539 (0.5) | 596 (0.5) | 5,727 (1.9) | 10 (1.7) | 8 (1.1) | 10 (1.4) |
|  | Cardiovascular Medicine | 604 (0.7) | 1,507 (1.5) | 1,882 (1.6) | 3,993 (1.3) | 31 (5.3) | 41 (5.9) | 42 (5.9) |
|  | Infectious Diseases | 523 (0.6) | 2,569 (2.5) | 698 (0.6) | 3,790 (1.2) | 6 (1.0) | 11 (1.6) | 9 (1.3) |
|  | Neurology | 882 (1.0) | 1,329 (1.3) | 1,524 (1.3) | 3,735 (1.2) | 17 (2.9) | 23 (3.3) | 21 (3.0) |
|  | Traditional Chinese Medicine | 284 (0.3) | 972 (1.0) | 2,337 (2.0) | 3,593 (1.2) | 15 (2.6) | 16 (2.3) | 20 (2.8) |
| Surgery | Sports Medicine | 5,411 (6.4) | 6,453 (6.4) | 14,177 (12.0) | 26,041 (8.6) | 35 (6.0) | 37 (5.3) | 42 (5.9) |
|  | Orthopedics | 4,323 (5.1) | 5,043 (5.0) | 6,527 (5.5) | 15,893 (5.2) | 48 (8.2) | 54 (7.7) | 56 (7.9) |
|  | General Surgery | 1,822 (2.1) | 2,749 (2.7) | 3,585 (3.0) | 8,156 (2.7) | 27 (4.6) | 30 (4.3) | 32 (4.5) |
|  | Urology | 684 (0.8) | 1,169 (1.2) | 1,056 (0.9) | 2,909 (1.0) | 21 (3.6) | 26 (3.7) | 24 (3.4) |
| Pediatrics | Pediatrics | 3,428 (4.0) | 6,192 (6.1) | 4,417 (3.7) | 14,037 (4.6) | 27 (4.6) | 30 (4.3) | 28 (4.0) |
| Ophthalmology & Otorhinolaryngology | Otolaryngology (E.N.T.) | 967 (1.1) | 1,884 (1.9) | 2,264 (1.9) | 5,115 (1.7) | 22 (3.8) | 26 (3.7) | 26 (3.7) |
|  | Ophthalmology | 693 (0.8) | 1,128 (1.1) | 1,192 (1.0) | 3,013 (1.0) | 32 (5.5) | 40 (5.7) | 35 (5.0) |
| Others | Others | 2,724 (3.2) | 4,501 (4.4) | 5,488 (4.7) | 12,713 (4.2) | 134 (23.0) | 169 (24.2) | 173 (24.5) |
