# Supplementary material for: Association Between Physician Communication Features and Patient Outcomes in Telemedicine: Retrospective Cross-Sectional Observational Study
Source: J Med Internet Res. 2026 Mar 26;28:e86977. doi: 10.2196/86977 (PMC13021109; doi:10.2196/86977)
Supplement: Multimedia Appendix 3 [file jmir-v28-e86977-s003.docx]

The impact of response modality on satisfaction without controlling inverse Mills ratio (N = 304,337 visits) at Peking University Third Hospital, Beijing, China (2021–2023).

|  | *review* |
| --- | --- |
| Group B (95% CI, *P* value) | 0.233 (0.016 to 0.450, *P*=.04) |
| Group C (95% CI, *P* value) | 0.203 (-0.006 to 0.413, *P*=.057) |
| Controls | Y |
| Physician FE | Y |
| Day-of-week FE | Y |
| Year-month FE | Y |
